# COVID-19 diagnosis and hospital admissions in Brazil: a countrywide survey (Covitel, 2022)

**DOI:** 10.1590/1980-549720240052

**Published:** 2024-11-22

**Authors:** Renato Teixeira, Sofia Reinach, Fátima Marinho, Pedro Hallal, Fernando César Wehrmeister, Eduardo Ribes Kohn, Érika Carvalho de Aquino, Pedro de Paula, Luciana Monteiro Vasconcelos Sardinha

**Affiliations:** I Universidade Federal de Minas Gerais, School of Medicine, Graduate Program in Public Health – Belo Horizonte (MG), Brazil.; II Vital Strategies – São Paulo (SP), Brazil.; III University of Illinois Urbana-Champaign, College of Applied Health Sciences – Urbana, Illinois, United States of America.; IV Universidade Federal de Pelotas, Graduate Program in Epidemiology – Pelotas (RS), Brazil.; V Universidade Federal de Pelotas, Graduate Program in Physical Education – Pelotas (RS), Brazil.

**Keywords:** Epidemiological surveillance, COVID-19 Testing, Adult, Brazil

## Abstract

**Objective:**

To estimate the prevalence of COVID-19 diagnosis and hospital admissions, and to evaluate their correlates in a nationwide Brazilian sample.

**Methods:**

A cross-sectional study was carried out with secondary data from the Telephone Survey of Risk Factors for Chronic Noncommunicable Diseases in Times of Pandemic – Covitel 2022. The Covitel study uses cluster sampling, carried out through random digit dialing on landlines and cell phones, among people aged 18 years or over. The outcome of the “diagnosis of COVID-19” was based on a self-report of a confirmed case through laboratory tests or medical diagnoses. Those who reported COVID-19 were asked about the need for hospital admission due to COVID-19. Independent variables included sex, age, level of education, region, comorbidity, private health insurance plan, self-rated health, and employment status. The odds ratio was estimated using logistic regression models considering the complex sample design.

**Results:**

From the sample of 9000 people, the prevalence of COVID-19 diagnosis was 25.4% (95%CI 23.8–27.1%), ranging from 23.0% (95%CI 20.0–26.3%) in the Northeast region to 28.5% (95%CI 25.3–31.7%) in the South region. Middle-aged adults (35–44 years old) had the highest prevalence of COVID-19 diagnosis. The higher the level of education, the higher the prevalence of COVID-19 diagnosis.

**Conclusion:**

The prevalence of COVID-19 diagnosis is markedly different from that of SARS-CoV-2 infection at the population level. Socioeconomic differences in access to testing are the likely explanation. Older adults and people with comorbidities were more likely to be admitted to hospital due to COVID-19 in Brazil.

## INTRODUCTION

The World Health Organization (WHO) declared on January 31, 2020 the pandemic caused by infections with the SARS-CoV-2 virus (COVID-19)^1^. In Brazil, in the years 2020 and 2021, COVID-19 proved to be the leading cause of death^2^, causing a significant increase in mortality from natural causes of 44% in 2021^3^, with approximately 520 thousand deaths higher than expected.

Socioeconomic factors were decisive for the spread of this virus in the Brazilian population^4^. Research results indicate that the difficulty in accessing information and the dissemination of controversial data delayed the fight against the pandemic^4,5,6^. Authors of a population-based study conducted in Brazil demonstrated higher infection rates when compared to those reported by the responsible agencies in the country^6^. According to the study, there is a prevalence of one reported case for every ten infected individuals (tested based on seroprevalence)^6^. Social differences in contagion were observed in Brazil, with indigenous and brown people being more likely to be infected with COVID-19 compared to white people, and a higher prevalence of contagion among the poorest individuals compared to people in the highest income quintile^6^.

Understanding the epidemiological aspects of COVID-19 in the Brazilian population is relevant for the assessment of the national health system in dealing with pandemics. Based on data from Covitel (*Inquérito Telefônico de Fatores de Risco para Doenças Crônicas não Transmissíveis em Tempos de Pandemia* – Telephone Survey of Risk Factors for Chronic Noncommunicable Diseases in Times of Pandemic), which is nationally representative,^7^ in the present study we aimed to estimate the prevalence of COVID-19 diagnoses and hospitalizations and to evaluate their correlates in a nationwide Brazilian sample.

## METHODS

A cross-sectional study was conducted using secondary data from the Telephone Survey of Risk Factors for Chronic Noncommunicable Diseases in Times of Pandemic — Covitel 2022. This questionnaire used a probabilistic sample of the population aged 18 years or older, representative for the Brazilian population. The Covitel project was approved by the Research Ethics Committee of the School of Physical Education of Universidade Federal de Pelotas (Opinion No. 5.125.635)^8^.

Data were collected between January and May 2022, obtained by an outsourced company specialized in conducting population studies and market research. A telephone register of landlines and cell phone lines was used, carried out using a random digit dialing (RDD) method. The distribution of direct distance dialing (DDD) codes was considered, obtaining a representative sample of each region and the country as a whole. Those interviewed by landlines were drawn before the interview based on the list of all the residents of the household aged at least 18 years, ranked in ascending order by age. In addition, in the case of cell phone lines, the person responsible for them was interviewed. The verbal informed consent of all individuals who agreed to participate in the research was considered. Quality control was carried out daily, by the company’s supervisory team responsible for the interviews, and weekly, by the Covitel research team.

Sample strata were based on data from the 2010 Brazilian census conducted by the Brazilian Institute of Geography and Statistics (IBGE). Stratifications were carried out based on geographical region (Northeast, North, Southeast, South, and Midwest), sex (men and women), age (18–34; 35–49; and 50 years or more), and level of education (0–11 years and 12 years or more of formal education). Adjustments were made for age (the age group of 18–19 years corresponds to 2/5 of the 15–19 year category of the IBGE) and level of education (the category of 0–11 years of study was created for those under high school in the IBGE categories)^7^.

Minimum strata were generated to:

a) macroregion (1,800 interviews per macroregion of the country);
b) type of telephone (4,500 interviews for each type);
c) sex (5,200 interviews with women and 3,800 with men);
d) age (2,250 interviews with individuals aged between 18 and 34 years; 3,670 with those aged between 35 and 49 years, and 3,080 with individuals aged 50 years and over); and
e) level of education: 5,200 interviews with individuals from 0 to 11 years of formal education and 3,800 with people from 12 years of formal education or more.

The present study used as outcomes the diagnosis confirmed by a test or health professional (no and yes) — based on the question “Did you have COVID-19 confirmed by a laboratory test or medical diagnosis?” —, and hospitalization for COVID-19 (no and yes), based on the question “Have you ever been hospitalized while having COVID-19?”. The outcomes “diagnosis of COVID-19” and “hospitalizations for COVID-19” considered the period of time from the first confirmed case in the country to the time of the respective interview. There was no distinction between laboratory tests and clinical diagnosis through the public or private system.

Exposure variables were:

a) sex;
b) region of the country;
c) age;
d) race/skin color;
e) level of education:
f) marital status;
g) work;
h) health insurance plan before the pandemic;
i) health insurance plan during the pandemic;
j) good self-rated health before the pandemic;
j) good self-rated health during the pandemic;
l) diabetes;
m) hypertension;
n) depression; and
o) obesity.

To estimate the prevalence, tabulations were used that considered the characteristics in relation to the outcome “COVID-19 confirmed by laboratory test or medical diagnosis” and the outcome “Hospitalization for COVID-19 among those diagnosed with the virus.” Conversely, to generate the odds ratio estimates, logistic regression models were used considering the outcome according to each of the stratifiers. All analyses were carried out considering complex samples, taking into account individual sample weights and strata by region, using the survey module for complex data in the Stata 17.0 software (Stata Corp., College Station, TX, USA). The prevalence estimates and their respective 95% confidence intervals were based on the binomial distribution.

## RESULTS

A total of 9000 individuals were interviewed, numerically distributed equally between regions of the country and telephone types. The final sample included 58.2% women, 43.0% people with white skin color, and 39.5% people with 12 years or more of formal education. We observed a prevalence of COVID-19 diagnosis of 25.5% (95%CI 23.8–27.1), a higher prevalence of disease diagnosis in the South region (28.5% [95%CI 25.5–31.7]), and a lower prevalence in the Northeast region (23.0% [95%CI 20.0–26.3]). In relation to age groups, the 35–44 age group had a higher prevalence of this outcome (30.3% [95%CI 27.3–33.6]). We verified a higher prevalence of COVID-19 among the most educated individuals, of whom the group of people with more than 12 years of formal education had 38.4% [95%CI 36.0–41.0] of cases of COVID-19 infection, with a 2.5-fold chance (95%CI 2.07–3.01) greater than those who studied between 0 and 8 years. Individuals who reported to be of white race/skin color had a prevalence of 27.9% [95%CI 25.5–30.3], while those who reported to be of Black race/skin color had 24.5% [95%CI 22.39–26.82] (Table 1).

**Table 1:** Prevalence of COVID-19, hospitalization (among COVID-19 cases), and odds ratio according to demographic variables. Covitel 2022.

Variables	Prevalence of COVID-19	Odds ratio	Prevalence of hospitalized patients	Odds ratio
Sex				
Women	25.5 [23.5–27.6]	1.01 [0.85–1.19]	5.8 [4.2–7.7]	0.67 [0.43–1.03]
Men	25.4 [23.0–27.9]	1	8.4 [6.3–11.1]	1
Region				
Midwest	28.1 [25.2–31.2]	1.31 [0.85–1.19]	8.2 [5.7–11.8]	1.37 [0.76–2.47]
Northeast	22.9 [19.9–26.3]	1	6.2 [4.1–9.3]	1
North	25.3 [22.3–28.6]	1.14 [0.89–1.45]	8.0 [4.9–12.7]	1.33 [0.67–2.61]
Southeast	25.5 [22.8–28.3]	1.15 [0.91–1.44]	6.1[4.1–9.0]	1 [0.54–1.83]
South	28.5 [25.5–31.7]	1.34 [1.06–1.69]	9.7 [6.4–14.5]	1.64 [0.87–3.09]
Age group				
18 to 24 years	28.1 [23.0–33.8]	2.09 [1.52–2.89]	2.3 [0.9–5.3]	0.1 [0.04–0.26]
25 to 34 years	26.9 [23.4–30.9]	1.98 [1.52–2.56]	4.8 [2.8–8.0]	0.21 [0.10–0.42]
35 to 44 years	30.3 [27.3–33.5]	2.33 [1.85–2.93]	5.1[3.2–7.9]	0.22 [0.12–0.42]
45 to 54 years	26.1 [23.1–29.2]	1.88 [1.49–2.39]	7.7 [4.5–12.7]	0.35 [0.17–0.70]
55 to 64 years	21.1 [18.0–24.6]	1.43 [1.1–1.87]	16.3 [10.3–24.5]	0.81 [0.41–1.57]
65 years and over	15.7 [13.5–18.2]	1	19.4 [13.6–26.9]	1
Race/Skin color				
White	27.9 [25.5–30.3]	1.19 [1–1.41]	6.0 [4.4–8.1]	0.71 [0.46–1.11]
Black	24.5 [22.4–26.8]	1	8.2 [6.2–10.9]	1
Others	19.1 [13.6–26.0]	0.73 [0.48–1.1]	3.4 [1.1–10.2]	0.4 [0.12–1.32]
Level of education (years)				
0 to 8	20.0 [17.6–22.6]	1	9.0 [6.5–12.5]	1
9 to 11	25.9 [23.3–28.8]	1.4 [1.14–1.73]	5.9 [4.1–8.4]	0.63 [0.38–1.07]
12 or more	100 [35.95–40.97]	2.5 [2.07–3.01]	5.6 [3.9–7.9]	0.59 [0.35–1]
Marital status				
Have a partner	29.3 [27.1–31.6]	1.47 [1.24–1.73]	8.4 [6.5–10.6]	1.56 [0.97–2.50]
Without a partner	22.2 [20.1–24.5]	1	5.5 [3.8–7.9]	1

People who worked before and during the COVID-19 pandemic had higher prevalence of diagnosis of infection (30.3% [95%CI 28.1–32.7]) by the virus when compared to those who worked before, but not during the pandemic (21.2% [95%CI 16.7–26.6), and those who had never worked (17.9% [95%CI 15.6–20.5). Conversely, the chance of infection with COVID-19 was 1.9 times (95%CI 1.31–2.81) higher in those who did not work before, but worked during the pandemic. People who lived with a partner had a higher prevalence of COVID-19 infection (29.3% [95%CI 27.1–31.6]) compared to those without partners (22.3% [95%CI 20.2–24.5]). Thus, people who lived with a partner were 1.5 times (95%CI 1.24–1.73) more likely to be infected with COVID-19 compared to those without partners. People with health insurance plan demonstrated a higher prevalence of COVID-19 diagnosis. This incidence was 32.3% [95%CI 29.8–34.8], while those without health insurance plan indicated 21.4% [95%CI 19.4–23.4] of a positive test for COVID-19. Likewise, those who answered that they had health insurance plan during the pandemic had a higher prevalence of this outcome (33.2% [30.6–35.9%]) compared to those without health insurance plan (20.9% [95%CI 19.1–22.9]). Those who had health insurance plan during the COVID-19 pandemic had a 1.9 times (95%CI 1.6–2.2) greater chance of being diagnosed with COVID-19 than those without health insurance plan during the pandemic (Table 2).

**Table 2:** Prevalence of COVID-19, hospitalization (among COVID-19 cases), and odds ratio according to variables related to work, access to private health, and self-rated health. Covitel 2022.

Variables	Prevalence of COVID-19	Odds ratio	Prevalence of hospitalized patients	Odds ratio
Work				
Worked before and during the pandemic	30.3 [28.1–32.7]	2.00 [0.64–2.43]	5.7 [4.3–7.5]	0.46 [0.28–0.76]
Worked before, but not during the pandemic	21.2 [16.7–26.6]	1.23 [0.88–1.73]	5.7 [2.9–10.7]	0.46 [0.21–1.03]
Did not work before, but worked during the pandemic	29.5 [22.8–37.2]	1.92 [1.31–2.81]	6.9 [2.9–15.8]	0.58 [0.21–1.57]
Had never worked	17.9 [15.6–20.5]	1	11.6 [8.0–16.4]	1
Health insurance plan – pre-pandemic				
No	21.4 [19.4–23.4]	1	8.5 [6.4–11.2]	1
Yes	32.2 [29.8–34.8]	1.78 [1.51–2.1]	5.5 [4.1–7.4]	0.63 [0.41–0.97]
Health insurance plan – during the pandemic				
No	20.9 [19.0–22.9]	1	8.1 [6.0–10.8]	1
Yes	33.2 [30.6–35.9]	1.90 [1.61–2.24]	5.98 [4.55–7.8.21]	0.72 [0.47–1.11]
Good self-rated health — pre-pandemic				
No	24.6 [21.8–27.7]	1	10.4 [7.5–14.2]	1
Yes	25.7 [23.9–27.6]	1.06 [0.88–1.28]	6.0 [5.0–7.8]	0.55 [0.35–0.87]
Good self-rated health — during the pandemic				
No	28.6 [26.0–31.3]	1	10.5 [8.1–13.6]	1
Yes	23.6 [21.7–25.6]	0.77 [0.65–0.91]	4.6 [3.3–6.4]	0.41 [0.26–0.64]

Regarding the outcome “hospitalization due to COVID-19,” the older the age, the greater the chance of hospitalization. This predominance was lower in the 18–24 age group (2.3% [95%CI 1.0–5.3%]) compared to the age group of 65 years or older (19.4% [95%CI 13.6–26.9]). Those who did not work had a higher prevalence of hospitalization (11.6% [8.0–16.4%]) compared to those who worked both periods (5.7% [4.3–7.5%]). According to the odds ratio analysis for hospitalization based on the presence or absence of diabetes, hypertension, and obesity, there was greater chance of hospitalization among those with exposure. Hypertensive and obese patients were twice as likely (2.03 [95%CI 1.27–3.22]) to be hospitalized when compared to non-hypertensive and non-obese individuals. Diabetics were 2.4 times more likely to be hospitalized for COVID-19 (2.44 [95%CI 1.33–4.48]) compared to individuals without a diagnosis of diabetes. Conversely, being diagnosed with depression did not increase the chance of hospitalization for COVID-19 (Tables 1, 2, and 3).

**Table 3:** Hospitalization for COVID-19 (among COVID-19 cases) and odds ratio according to the evaluated comorbidities. Covitel 2022.

Variables	Prevalence of hospitalized patients	Odds ratio
Hypertension		
No	5.8 [4.5–7.6]	1
Yes	11.2 [7.9–15.4]	2.03 [1.27–3.22]
Diabetes		
No	6.3 [5.1–7.9]	1
Yes	14.3 [8.7–22.5]	2.44 [1.33–4.48]
Depression		
No	6.9 [5.6–8.7]	1
Yes	7.6 [4.4–12.9]	1.10 [0.59–2.06]
Obesity		
No	5.7 [4.4–7.4]	1
Yes	10.9 [7.8–15.1]	2.03 [1.27–3.22]

## DISCUSSION

The estimated prevalence of confirmed COVID-19 infection was 25.5%, and the estimated hospitalization was 7% among those diagnosed with the virus. White people, individuals with more years of formal education, people who had health insurance plan, and those who worked during the pandemic had higher prevalence of COVID-19 diagnosis in Brazil. Having 12 or more years of formal education increased the chance of being diagnosed with COVID-19 by 2.5 times compared to those who had up to 8 years of formal education. Those who did not work before, but started working during the pandemic, were 1.9 times more likely to be diagnosed with the virus. Older people and individuals with certain types of comorbidities had a greater chance of being hospitalized as a result of coronavirus infection. For example, having diabetes (2 times), high blood pressure (2.4 times), and obesity (2.4 times) made individuals more likely to be infected with COVID-19.

Access to the diagnosis seems to have influenced the estimates of the prevalence of COVID-19. In this study we show a greater tendency to test for coronavirus among people with greater financial capacity. To illustrate that we can mention the higher prevalence of COVID-19 among people with health insurance plans, people who had jobs, and people with more years of formal education. The insufficient availability of free COVID-19 tests for the country’s population size^9,10^ and the cost of testing at private laboratories may have been factors affecting testing among the poorest people^11^. At the end of the pandemic, Brazil had approximately 38 million confirmed cases based on official government data^12^; however, already in 2020, studies indicated an underreporting of COVID-19 cases in the country^6^.

According to data from the EPICOVID study in Brazil, there is a higher prevalence in subgroups other than those observed in the present study^6^. Indigenous and brown people and those in the poorest income quintile had higher prevalence of COVID-19^6^. Methodological differences may explain the conflicting results between the two studies, reinforcing the idea of inequality in testing. When measured by serological test^6^, poorer population groups had a higher prevalence of COVID-19, while population groups with greater socioeconomic status had a higher prevalence of the virus according to data obtained by prior testing in the Covitel study. This result indicates that underreporting may have been even more pronounced among people in conditions of socioeconomic vulnerability.

Authors of studies conducted in other countries show similar inequalities in terms of testing for COVID-19. There is evidence that neighborhoods composed of people with greater purchasing power have greater distribution of tests, even in high-income countries^13,14,15^. Testing is important for interrupting the chain of transmission of the virus^16^, in such a way that inequalities in carrying it out may represent an increase in contagion in specific groups and, as a result, a higher mortality among disadvantaged classes^15,17^ With the proper application of the tests, isolation measures, such as quarantine, could have resulted in a greater impact in interrupting the transmission of the virus^18^.

The lethality of the disease may vary according to age and certain clinical conditions, such as: advanced age, chronic obstructive pulmonary disease, asthma and oxygen dependency, serious heart problems, uncontrolled hypertension, diabetes mellitus, chromosomal disorders or immunosuppression, kidney failure, high-risk pregnancy, severe obesity (Body Mass Index – BMI>40), and liver diseases. These conditions aggravate the disease and make it necessary to stay in Intensive Care Units (ICUs)^19^.

Some comorbidities, such as obesity, high blood pressure, and diabetes mellitus, are considered risk factors for cases of severe COVID-19^20,21^. Diabetes and hypertension are the most reported types in patients with severe coronavirus infection^22,23^ In this study we showed that hypertensive patients diagnosed with this virus were twice as likely to be hospitalized as those without hypertension. Diabetics were 2.4 times more likely than non-diabetics. This result reinforces the idea of the severity of COVID-19 among those with diabetes and hypertension.

Obesity is considered a risk factor for several chronic noncommunicable diseases and is associated with a series of health issues, including diabetes and hypertension. In this study, obese people were twice as likely to be hospitalized as those who were not obese. Obesity is a comorbidity that is increasing in Brazil, creating an urgent need for discussion so that combat measures can be implemented^24^. Our results corroborate the findings of other patients who have indicated that obesity is an aggravating factor in the hospitalization of COVID-19 cases^25^.

This study has some methodological limitations that must be considered when interpreting its results. This is a survey that measured the prevalence of COVID-19 based on interviews based on prior diagnosis by laboratory test or medical examination. Inequality in testing and access to medical diagnosis may underestimate the prevalence among the most economically disadvantaged people to a greater extent. We could not differentiate whether the diagnosis of COVID-19 was made by tests or clinical evaluation, and whether it was performed in a public or private service. Data on depression and self-rated health may present reverse causality. Although there is difficulty in estimating the actual prevalence of COVID-19, the present research has methodological characteristics that allow us to represent the entire adult population (18 years or older) in Brazil and to identify the epidemiological aspects of COVID-19 — as well as such inequalities at the national level.

Therefore, we conclude that in this study we indicated the possibility of inequalities in people’s access to testing during the COVID-19 pandemic. The lack of tests in the public service during the critical phase of the pandemic and the unequal access to COVID-19 tests posed risks for some population subgroups. Measures, such as isolation of positive cases and quarantine of suspected cases, may have been hampered by the lack of testing. Adequate financial investment must be guaranteed by the Federal Government, so as to enable equitable access to health care provided by the Brazilian Unified Health System.
