# Supplementary material for: COVID-19 diagnosis and hospital admissions in Brazil: a countrywide survey (Covitel, 2022)
Source: Rev Bras Epidemiol. 2024 Nov 22;27:e240052. doi: 10.1590/1980-549720240052 (PMC11653953; doi:10.1590/1980-549720240052)
Supplement: Translation

# Translation: Diagnóstico de COVID-19 e internações hospitalares: um estudo nacional (Covitel, 2022)

**DOI:** 10.1590/1980-549720240052.2

**Authors:** Renato Teixeira, Sofia Reinach, Fátima Marinho, Pedro Hallal, Fernando César Wehrmeister, Eduardo Ribes Kohn, Érika Carvalho de Aquino, Pedro de Paula, Luciana Monteiro Vasconcelos Sardinha

**Affiliations:** I Universidade Federal de Minas Gerais, School of Medicine, Graduate Program in Public Health – Belo Horizonte (MG), Brasil.; II Vital Strategies – São Paulo (SP), Brasil.; III University of Illinois Urbana-Champaign, College of Applied Health Sciences – Urbana, Illinois, Estados Unidos.; IV Universidade Federal de Pelotas, Graduate Program in Epidemiology – Pelotas (RS), Brasil.; V Universidade Federal de Pelotas, Graduate Program in Physical Education – Pelotas (RS), Brasil.

**Keywords:** COVID-19, Teste para COVID-19, Adulto, Brasil

## Abstract

**Objetivo:**

Estimar a prevalência de diagnóstico e internações hospitalares por COVID-19 e avaliar seus correlatos entre adultos no Brasil.

**Métodos:**

Um estudo transversal, com dados secundários do Inquérito Telefônico de Fatores de Risco para Doenças Crônicas não Transmissíveis em tempos de Pandemia — Covitel 2022 foi realizado. O estudo Covitel utiliza amostragem por conglomerados, realizada a partir de discagem aleatória de dígitos para linhas telefônicas fixas e celulares, entre pessoas de 18 anos ou mais. O desfecho “diagnóstico de COVID-19” foi baseado no autorrelato de um caso confirmado, por meio de exames laboratoriais ou diagnósticos médicos. Para aqueles que relataram COVID-19, foi questionado sobre a necessidade de internação hospitalar devido à COVID-19. As variáveis independentes incluíram sexo, idade, escolaridade, região, comorbidade, plano de saúde privado, autoavaliação de saúde e situação profissional. A razão de chances foi estimada por regressão logística considerando o desenho amostral complexo.

**Resultados:**

A partir da amostra de 9000 pessoas obteve-se prevalência de diagnóstico de COVID-19 de 25,4% (IC95% 23,8–27,1%), variando de 23,0% (IC95% 20,0–26,3%) na região Nordeste a 28,5% (IC95% 25,3–31,7%) na região Sul. Adultos entre 35–44 anos tiveram a maior prevalência de diagnóstico de COVID-19. Quanto maior a escolaridade, maior foi a prevalência do diagnóstico de COVID-19.

**Conclusão:**

A prevalência do diagnóstico de COVID-19 é marcadamente diferente daquela da infecção por SARS-CoV-2 a nível populacional. As diferenças socioeconômicas no acesso aos testes são a explicação provável. Idosos e pessoas com comorbidades tiveram maior probabilidade de serem internados em hospital devido à COVID-19 no Brasil.

## INTRODUÇÃO

A Organização Mundial da Saúde (OMS) declarou em 31 de janeiro de 2020 a pandemia causada por infecções pelo vírus SARS-CoV-2 (COVID-19)^1^. No Brasil, nos anos de 2020 e 2021, a COVID-19 se mostrou como a principal causa de morte^2^, causando um significativo aumento de mortalidade por causas naturais de 44% em 2021^3^, sendo aproximadamente 520 mil óbitos acima do esperado.

Os fatores socioeconômicos foram determinantes para o avanço desse vírus na população brasileira^4^. Resultados de pesquisas indicam que a dificuldade de acesso a informação e disseminação de dados controversos atrasaram o combate à pandemia^4,5,6^. Um estudo de base populacional no Brasil demonstrou taxas de contágio superiores quando comparadas às notificadas pelos órgãos responsáveis no país^6^. O estudo indica prevalência de um caso notificado para cada dez indivíduos infectados (testado a partir de soroprevalência)^6^. Diferenças sociais no contágio foram observadas no Brasil, com mais chances de pessoas das etnias indígena e parda se infectarem por COVID-19 em comparação às pessoas de cor de pele branca, e maior prevalência de contágio entre os mais pobres em relação às pessoas no quintil de renda mais alto^6^.

Compreender os aspectos epidemiológicos da COVID-19 na população brasileira é relevante para a avaliação do sistema de saúde nacional no enfrentamento de pandemias. Baseado em dados do inquérito Covitel (Inquérito Telefônico de Fatores de Risco para Doenças Crônicas não Transmissíveis em Tempos de Pandemia), que possui representatividade nacional,^7^ o presente estudo tem como objetivo estimar a prevalência de diagnóstico e internações hospitalares por COVID-19 e avaliar seus correlatos em uma amostra nacional brasileira.

## MÉTODOS

Foi feito um estudo transversal utilizando os dados secundários provenientes do Inquérito Telefônico de Fatores de Risco para Doenças Crônicas não Transmissíveis em Tempos de Pandemia — Covitel 2022. Esse questionário utilizou amostra probabilística da população com idade de 18 anos ou mais, representativa para a população brasileira. O projeto Covitel foi aprovado pelo Comitê de Ética em Pesquisa da Escola Superior de Educação Física da Universidade Federal de Pelotas (Parecer número 5.125.635)^8^.

Os dados foram coletados no período entre janeiro e maio de 2022, obtidos por uma empresa terceirizada especializada na condução de estudos populacionais e pesquisas de mercado. Foi utilizado um cadastro telefônico de linhas residenciais e de celular, realizado por método de discagem aleatória de dígitos (RDD). Foi considerada a distribuição dos códigos de discagem direta à distância (DDD), obtendo amostra representativa de cada região e do país em sua totalidade. Os entrevistados em linhas de telefone fixo foram sorteados antes da entrevista com base na relação de todos os moradores do domicílio com pelo menos 18 anos, ordenados de forma crescente por idade. Ademais, no caso das linhas de celular, foi entrevistado o responsável por ela. Foi considerado o consentimento livre e esclarecido (TCLE) verbal de todos os indivíduos que concordaram em participar da pesquisa. O controle de qualidade foi realizado diariamente, pela equipe de supervisão da empresa responsável pelas entrevistas, e semanalmente, pela equipe de pesquisa do Covitel.

Estratos amostrais foram baseados nos dados do censo brasileiro de 2010 realizado pelo Instituto Brasileiro de Geografia e Estatística (IBGE). Foram realizadas estratificações com base na região geográfica (Nordeste, Norte, Sudeste, Sul e Centro-oeste), no sexo (masculino e feminino), na idade (18–34; 35–49 e 50 anos ou mais) e na escolaridade (0–11 anos e 12 anos ou mais de escolaridade). Foram feitas adaptações para idade (a faixa etária de 18–19 anos corresponde a 2/5 da categoria de 15–19 anos do IBGE) e escolaridade (foi criada a categoria de 0–11 anos de estudo para quem estava abaixo do ensino médio nas categorias do IBGE)^7^.

Estratos mínimos foram gerados para:

a) macrorregião (1.800 entrevistas por macrorregião do país);
b) tipo de telefone (4.500 entrevistas para cada tipo);
c) sexo (5.200 entrevistas com mulheres e 3.800 com homens);
d) idade (2.250 indivíduos entre 18 e 34 anos, 3.670 com aqueles entre 35 e 49 anos e 3.080 com pessoas de 50 anos ou mais) e;
e) escolaridade: 5.200 entrevistas com indivíduos de 0 a 11 anos de estudo e 3.800 com pessoas de 12 anos de estudo ou mais.

O presente estudo utilizou como desfechos o diagnóstico confirmado por teste ou profissional da saúde (não e sim) — baseado na pergunta “O(a) Sr(a) teve COVID-19 confirmada por teste laboratorial ou diagnóstico médico?” —, e internação por COVID-19 (não e sim), fundamentado na pergunta “Em alguma das vezes que teve COVID-19, o(a) sr(a) foi hospitalizado(a)?”. Os desfechos “diagnóstico de COVID-19” e “internações por COVID-19” consideraram o período de tempo desde o primeiro caso confirmado no país até o momento de realização da respectiva entrevista. Não houve distinção entre testes laboratoriais e diagnóstico clínico por meio do sistema público ou privado.

Variáveis de exposição foram:

a) sexo;
b) região do país;
c) idade;
d) raça/cor;
e) escolaridade;
f) situação conjugal;
g) trabalho;
h) plano de saúde antes da pandemia;
i) plano de saúde durante a pandemia;
j) autoavaliação de saúde boa antes da pandemia;
k) autoavaliação de saúde boa durante a pandemia;
l) diabetes;
m) hipertensão;
n) depressão e,
o) obesidade.

Para estimar as prevalências, foram utilizadas tabulações que consideraram as características em relação ao desfecho “COVID-19 confirmada por teste laboratorial ou diagnóstico médico” e ao desfecho “Internação por COVID-19 entre os diagnosticados com o vírus”. Em contrapartida, para gerar as estimativas de razão de odds, foram utilizados modelos de regressão logística considerando o desfecho de acordo com cada um dos estratificadores. Todas as análises foram realizadas considerando amostras complexas, tendo em conta os pesos amostrais individuais e os estratos por regiões, utilizando o módulo *survey* para dados complexos no software *Stata 17.0* (Stata Corp., College Station, TX, USA). As estimativas de prevalências e seus respectivos intervalos de confiança de 95% foram baseadas na distribuição binomial.

## RESULTADOS

Foram entrevistados 9000 indivíduos, distribuídos numericamente de forma igual entre as regiões do país e tipos de telefone. A amostra final incluiu 58,2% mulheres, 43,0% pessoas com cor de pele branca, 39,5% pessoas com 12 anos ou mais de escolaridade. Observou-se uma prevalência de diagnóstico de COVID-19 de 25,5% (IC95% 23,8–27,1), bem como foi observada maior prevalência de diagnóstico da doença na região Sul (28,5% [IC95% 25,5–31,7]), e menor prevalência na região Nordeste (23,0% [IC95% 20,0–26,3]). Em relação aos grupos etários, a faixa de 35 a 44 anos teve maior prevalência desse desfecho (30,3% [IC95% 27,3–33,6]). Foi observada uma maior prevalência de COVID-19 entre os mais escolarizados, dos quais o grupo de pessoas com mais de 12 anos de estudo apresentou 38,4% [IC95% 36,0–41,0] de casos de infecção por COVID-19, com uma chance 2,5 vezes (IC95% 2,07–3,01) maior do que as pessoas que estudaram entre 0 e 8 anos. Aquelas pessoas que se denominaram da raça/cor branca apresentaram uma prevalência de 27,9% [IC95% 25.5–30.3], enquanto os autodeclarados de raça/cor preta tiveram 24,5% [IC95% 22,39–26,82] (Tabela 1).

**Tabela 1:** Prevalência de COVID-19, internação (entre os casos de COVID-19) e razão de odds segundo variáveis demográficas. Covitel 2022.

Variáveis	Prevalência de COVID	Razão de odds	Prevalência de internados	Razão de odds
Sexo				
Feminino	25.5 [23.5–27.6]	1.01 [0.85–1.19]	5.8 [4.2–7.7]	0.67 [0.43–1.03]
Masculino	25.4 [23.0–27.9]	1	8.4 [6.3–11.1]	1
Região				
Centro-oeste	28.1 [25.2–31.2]	1.31 [0.85–1.19]	8.2 [5.7–11.8]	1.37 [0.76–2.47]
Nordeste	22.9 [19.9–26.3]	1	6.2 [4.1–9.3]	1
Norte	25.3 [22.3–28.6]	1.14 [0.89–1.45]	8.0 [4.9–12.7]	1.33 [0.67–2.61]
Sudeste	25.5 [22.8–28.3]	1.15 [0.91–1.44]	6.1[4.1–9.0]	1 [0.54–1.83]
Sul	28.5 [25.5–31.7]	1.34 [1.06–1.69]	9.7 [6.4–14.5]	1.64 [0.87–3.09]
Faixa etária				
18 a 24	28.1 [23.0–33.8]	2.09 [1.52–2.89]	2.3 [0.9–5.3]	0.1 [0.04–0.26]
25 a 34	26.9 [23.4–30.9]	1.98 [1.52–2.56]	4.8 [2.8–8.0]	0.21 [0.10–0.42]
35 a 44	30.3 [27.3–33.5]	2.33 [1.85–2.93]	5.1[3.2–7.9]	0.22 [0.12–0.42]
45 a 54	26.1 [23.1–29.2]	1.88 [1.49–2.39]	7.7 [4.5–12.7]	0.35 [0.17–0.70]
55 a 64	21.1 [18.0–24.6]	1.43 [1.1–1.87]	16.3 [10.3–24.5]	0.81 [0.41–1.57]
65 e mais	15.7 [13.5–18.2]	1	19.4 [13.6–26.9]	1
Raça/cor				
Branca	27.9 [25.5–30.3]	1.19 [1–1.41]	6.0 [4.4–8.1]	0.71 [0.46–1.11]
Preta	24.5 [22.4–26.8]	1	8.2 [6.2–10.9]	1
Outras	19.1 [13.6–26.0]	0.73 [0.48–1.1]	3.4 [1.1–10.2]	0.4 [0.12–1.32]
Escolaridade (anos)				
0 a 8	20.0 [17.6–22.6]	1	9.0 [6.5–12.5]	1
9 a 11	25.9 [23.3–28.8]	1.4 [1.14–1.73]	5.9 [4.1–8.4]	0.63 [0.38–1.07]
12 ou mais	100 [35.95–40.97]	2.5 [2.07–3.01]	5.6 [3.9–7.9]	0.59 [0.35–1]
Situação conjugal				
Com companheiro(a)	29.3 [27.1–31.6]	1.47 [1.24–1.73]	8.4 [6.5–10.6]	1.56 [0.97–2.50]
Sem companheiro(a)	22.2 [20.1–24.5]	1	5.5 [3.8–7.9]	1

As pessoas que trabalhavam antes e durante a pandemia de COVID-19 tiveram prevalências mais altas de diagnóstico de infecção (30,3% [IC95% 28,1–32,7]) pelo vírus quando comparadas aos que trabalhavam antes, mas não durante a pandemia (21,2% [IC95% 16,7–26,6), e aos que nunca trabalharam (17,9% [IC95% 15,6–20,5). Por outro lado, a chance de infecção por COVID-19 foi 1,9 vezes (IC95% 1,31–2,81) maior em quem não trabalhava anteriormente, mas trabalhou durante a pandemia. Pessoas que viviam com companheiro apresentaram uma prevalência maior de infecção por COVID-19 (29,3% [IC95% 27,1–31,6]) em relação às sem companheiros (22,3% [IC95% 20,2–24,5]). Dessa forma, pessoas que viviam com companheiro tiveram 1,5 vezes (IC95% 1,24–1,73) mais chance de infecção por COVID-19 em comparação às sem companheiros. Pessoas com plano de saúde demonstraram prevalência superior de diagnóstico de COVID-19. Essa incidência foi de 32,3% [IC95% 29,8–34,8], enquanto quem não possuía plano de saúde indicou 21,4% [IC95% 19,4–23,4] de testagem positiva para COVID-19. Da mesma forma, quem respondeu que possuía plano de saúde durante a pandemia teve maior prevalência desse desfecho (33,2% [30,6–35,9%]) em relação àqueles sem plano de saúde (20,9% [IC95% 19,1–22,9]). Aqueles que possuíam plano de saúde durante a pandemia de COVID-19 tiveram uma chance de ser diagnosticado com COVID-19 de 1.9 vezes (IC95% 1.6–2.2) maior do que quem não tinha plano de saúde durante a pandemia (Tabela 2).

**Tabela 2:** Prevalência de COVID-19, internação (entre os casos de COVID-19) e razão de odds segundo variáveis relacionadas ao trabalho, acesso a saúde privada e autopercepção de saúde. Covitel 2022.

Variáveis	Prevalência de COVID	Razão de odds	Prevalência de internados	Razão de odds
Trabalho				
Trabalhava antes e durante a pandemia	30.3 [28.1–32.7]	2.00 [0.64–2.43]	5.7 [4.3–7.5]	0.46 [0.28–0.76]
Trabalhava antes, mas não durante a pandemia	21.2 [16.7–26.6]	1.23 [0.88–1.73]	5.7 [2.9–10.7]	0.46 [0.21–1.03]
Não trabalhava, mas trabalhou durante a pandemia	29.5 [22.8–37.2]	1.92 [1.31–2.81]	6.9 [2.9–15.8]	0.58 [0.21–1.57]
Nunca trabalhou	17.9 [15.6–20.5]	1	11.6 [8.0–16.4]	1
Possuir plano de saúde – pré-pandemia				
Não	21.4 [19.4–23.4]	1	8.5 [6.4–11.2]	1
Sim	32.2 [29.8–34.8]	1.78 [1.51–2.1]	5.5 [4.1–7.4]	0.63 [0.41–0.97]
Possuir plano de saúde – durante pandemia				
Não	20.9 [19.0–22.9]	1	8.1 [6.0–10.8]	1
Sim	33.2 [30.6–35.9]	1.90 [1.61–2.24]	5.98 [4.55–7.8.21]	0.72 [0.47–1.11]
Boa percepção da saúde – pré-pandemia				
Não	24.6 [21.8–27.7]	1	10.4 [7.5–14.2]	1
Sim	25.7 [23.9–27.6]	1.06 [0.88–1.28]	6.0 [5.0–7.8]	0.55 [0.35–0.87]
Boa percepção da saúde – durante-pandemia				
Não	28.6 [26.0–31.3]	1	10.5 [8.1–13.6]	1
Sim	23.6 [21.7–25.6]	0.77 [0.65–0.91]	4.6 [3.3–6.4]	0.41 [0.26–0.64]

Em relação ao desfecho “internação em decorrência da COVID-19”, quanto maior a idade, maior a chance de hospitalização. Essa preponderância foi menor na faixa etária de 18 a 24 anos (2,3% [IC95% 1,0–5,3%]) se comparada à faixa etária de 65 anos ou mais (19,4% [IC95% 13,6–26,9]). Aqueles que não trabalharam apresentaram maior prevalência de internação (11,6% [8,0–16,4%]) em relação aos que trabalharam nos dois momentos (5,7% [4,3–7,5%]). A análise de razão de *odds* para internação com base na presença ou ausência de diabetes, hipertensão e obesidade mostrou maior chance de internação entre quem possui a exposição. Os hipertensos e os obesos tiveram duas vezes mais chance (2.03 [IC95% 1.27–3.22]) de serem internados quando comparados aos não-hipertensos e os não-obesos. Os diabéticos tiveram 2.4 vezes mais chance de serem internados por COVID-19 (2.44 [IC95% 1.33–4.48]) em relação aos indivíduos sem diagnóstico de diabetes. Em contrapartida, ter diagnóstico de depressão não aumentou a chance de internação por COVID-19 (Tabela 1, 2 e 3).

**Tabela 3:** Internação por COVID-19, (entre os casos de COVID-19) e razão de odds segundo comorbidades avaliadas. Covitel 2022.

Variáveis	Prevalência de internados	Razão de odds
Hipertensão		
Não	5.8 [4.5–7.6]	1
Sim	11.2 [7.9–15.4]	2.03 [1.27–3.22]
Diabetes		
Não	6.3 [5.1–7.9]	1
Sim	14.3 [8.7–22.5]	2.44 [1.33–4.48]
Depressão		
Não	6.9 [5.6–8.7]	1
Sim	7.6 [4.4–12.9]	1.10 [0.59–2.06]
Obesidade		
Não	5.7 [4.4–7.4]	1
Sim	10.9 [7.8–15.1]	2.03 [1.27–3.22]

## DISCUSSÃO

A estimativa de prevalência de infecção confirmada por COVID-19 foi de 25,5%, e a estimativa de internação foi de 7% entre os diagnosticados com o vírus. Pessoas de cor de pele branca, pessoas com mais anos de estudo, pessoas que possuíam plano de saúde e os que possuíam trabalho durante a pandemia tiveram maiores prevalências de diagnóstico de COVID-19 no Brasil. Possuir 12 ou mais anos de escolaridade aumentou em 2.5 vezes a chance de ser diagnosticado com COVID-19 em comparação a quem teve até 8 anos de estudo. Quem não trabalhava antes, mas começou a trabalhar durante a pandemia teve 1.9 vezes mais chance de ser diagnosticado com o vírus. Pessoas idosas e pessoas com alguns tipos de comorbidades tiveram uma chance maior de serem internados em decorrência da infecção pelo coronavírus. Por exemplo, ser portador de diabetes (2 vezes), de hipertensão (2.4 vezes), e obesidade (2.4 vezes) mais chance de ser infectado por COVID-19.

O acesso ao diagnóstico parece ter influenciado as estimativas de prevalência da COVID-19. O presente estudo apresenta uma tendência de testagem para coronavírus maior entre as pessoas com maior capacidade financeira. Um exemplo disso foram as prevalências mais elevadas de COVID-19 entre as pessoas com planos de saúde, pessoas que tinham emprego e pessoas com mais anos de estudo. A disponibilidade de testes de COVID-19 gratuitos foi insuficiente para o tamanho populacional do país^9,10^ e o custo dos testes em laboratórios privados podem ter sido fatores que afetaram a testagem entre as pessoas mais pobres^11^. Ao final da pandemia, o Brasil contabilizou aproximadamente 38.000.000 casos confirmados baseados em dados oficiais do governo^12^, entretanto, já em 2020, estudos indicavam uma subnotificação dos casos de COVID-19 no país^6^.

Dados do estudo EPICOVID no Brasil mostram prevalências mais altas em subgrupos diferentes dos observados no presente estudo^6^. Pessoas das etnias indígena e parda e as pessoas no quintil de renda mais pobre apresentaram prevalências mais altas de COVID-19^6^. Diferenças metodológicas podem explicar os resultados conflitantes entre os dois estudos, reforçando a ideia de desigualdade na testagem. Quando medido por teste sorológico^6^, grupos populacionais mais pobres tiveram maior prevalência de COVID-19, enquanto grupos populacionais com maior capacidade socioeconômica tiveram maior prevalência do vírus de acordo com os dados obtidos por testagem prévia no estudo Covitel. Esse resultado indica que a subnotificação pode ter sido ainda mais acentuada entre pessoas em condições de vulnerabilidade socioeconômica.

Estudos em outros países apresentam desigualdades semelhantes em termos de testagem para COVID-19. Evidências mostram bairros formado por pessoas com maior poder aquisitivo tendo maior distribuição de testes, ainda que em países de alta renda^13,14,15^. A testagem é importante para a interrupção da cadeia de transmissão do vírus^16^, portanto, desigualdades na realização desta podem representar aumento do contágio em grupos específicos e, em decorrência, uma maior mortalidade entre classes desfavorecidas^15,17^. Com a aplicação adequada dos testes, as medidas de isolamento, como a quarentena, poderiam ter resultado em um maior impacto na interrupção da transmissão do vírus^18^.

A letalidade da doença pode variar de acordo com a faixa etária e segundo determinadas condições clínicas, tais como: idade avançada, doença pulmonar obstrutiva crônica, asma e dependência de oxigênio, problemas cardíacos graves, hipertensão não controlada, diabetes *mellitus*, doenças cromossômicas ou imunossupressão, insuficiência renal, gestação de alto risco, obesidade grave (Índice de Massa Corporal — IMC>40) e doenças hepáticas. Essas condições agravam a doença e tornam necessária a internação em Unidades de Terapia Intensiva (UTI)^19^.

Algumas comorbidades, como obesidade, hipertensão arterial e Diabetes *Mellitus*, são consideradas fatores de risco para casos de COVID-19 grave^20,21^. O diabetes e a hipertensão são os tipos mais relatados em pacientes com infecção grave^22,23^ pelo conronavírus. O presente estudo mostrou que os hipertensos diagnosticados com esse vírus tiveram duas vezes mais chance de internação em relação àqueles que não tem hipertensão. Os diabéticos tiveram 2,4 vezes mais chance em relação aos não-diabéticos. Esse resultado reforça a ideia de gravidade da COVID -19 entre aqueles que tem diabetes e hipertensão.

A obesidade é considerada fator de risco para diversas doenças crônicas não transmissíveis e está associada a uma série de danos à saúde, entre eles diabetes e hipertensão. Nesta pesquisa, os obesos tiveram duas vezes mais chance de internação em relação aos não-obesos. A obesidade é uma comorbidade que está aumentando no Brasil, gerando a necessidade urgente de discussão para que medidas de combate possam ser realizadas^24^. Os resultados encontrados nesse estudo corroboram com os achados de outros tratados que têm indicado que a obesidade é um fator agravante na hospitalização de casos de COVID-19^25^.

Este estudo apresenta algumas limitações metodológicas que devem ser consideradas na interpretação de seus resultados. Trata-se de um inquérito que mediu a prevalência de COVID-19 com base em entrevistas fundamentadas no diagnóstico prévio por teste laboratorial ou exame médico. A desigualdade na testagem e no acesso ao diagnóstico médico podem subestimar em maior grau a prevalência entre as pessoas mais desfavorecidas economicamente. Não foi possível diferenciar se o diagnóstico de COVID-19 foi realizado por testes ou avaliação clínica, e se foi realizado em serviço público ou privado. Dados de depressão e autoavaliação de saúde podem apresentar causalidade reversa. Embora exista dificuldade em estimar a prevalência real de COVID-19, a presente pesquisa possui características metodológicas que permitem representar toda população adulta (18 anos ou mais) do Brasil e identificar os aspectos epidemiológicos da COVID-19, bem como tais desigualdades em âmbito nacional.

Conclui-se, portanto, que este estudo indica a possibilidade de desigualdades no acesso à testagem de pessoas durante a pandemia de COVID-19. A falta de testes no serviço público durante a fase crítica da pandemia e o acesso desigual a testes de COVID-19 representaram riscos para alguns subgrupos populacionais. Medidas como isolamento de casos positivos e quarentena dos casos suspeitos podem ter sido prejudicadas pela falta de testagem. O investimento financeiro adequado deve ser garantido pelo Governo Federal, de modo que possibilite o acesso equitativo aos cuidados de saúde previsto pelo Sistema Único de Saúde do Brasil.
